# Structure, clustering and functional insights of repeats configurations in the upstream promoter region of the human coding genes

**DOI:** 10.1186/s12864-018-5196-6

**Published:** 2018-12-11

**Authors:** Fabian Tobar-Tosse, Patricia E. Veléz, Eliana Ocampo-Toro, Pedro A. Moreno

**Affiliations:** 1 Departamento de Ciencias Básicas de la Salud, Pontificia Universidad Javeriana Cali, Cali, Colombia; 2 Departamento de Biología, FACNED, Universidad del Cauca, Popayán, Colombia; 3 Especialización en Hematología y Oncología Clínica, Universidad Libre Seccional Cali, Cali, Colombia; 4 Escuela de Ingeniería de Sistemas y Computación, Universidad del Valle, Cali, Colombia

**Keywords:** Repetitive DNA sequences, Upstream promoter region, Human coding genes, Cell signaling pathway, Functional enrichment

## Abstract

**Background:**

Repetitive DNA sequences (Repeats) are significant regions in the human genome that have a specific genomic distribution, structure, and several binding sites for genome architecture and function. In consequence, the possible configurations of Repeats in specific and dynamic regions like the gene promoters could define footprints for molecular mechanisms, pathways, and cell function beyond their density in the genome. Here we explored the distribution of Repeats in the upstream promoter region of the human coding genes with the aim to identify specific configurations, clusters and functional meaning of those elements. Our method includes structural descriptions, hierarchical clustering, pathway association, and functional enrichment analysis.

**Results:**

We report here several configurations of Repeats in the upstream promoter region (UPR), which define 2729 patterns for the 80% of the human coding genes. There are 47 types of Repeats in these configurations, where the most frequent were Alu, Low_complexity, MIR, Simple_repeat, LINE/L2, LINE/L1, hAT-Charlie, and ERV1. The distribution, length, and the high frequency of Repeats in the UPR defines several patterns and clusters, where the minimum frequency of configuration among Repeats was higher than 0.7. We found those clusters associated with cellular pathways and ontologies; thus, it was plausible to determine groups of Repeats to specific functional insights, for example, pathways for Genetic Information Processing or Metabolism shows particular groups of Repeats with specific configurations.

**Conclusion:**

Based on these findings, we propose that specific configurations of repetitive elements describe frequent patterns in the upstream promoter for sets of human coding genes, which those correlated to specific and essential cell pathways and functions.

**Electronic supplementary material:**

The online version of this article (10.1186/s12864-018-5196-6) contains supplementary material, which is available to authorized users.

## Background

The broad repertoire of repetitive DNA sequences “Repeats” in the human genome has defined properties for the genome structure and organization, mainly associated to transposable elements (TEs), whose high density involves a direct connection with regulatory footprints. For instance, LINEs as the densest Repeat in the human genome [1], and SINEs as the most common element in gene promoters [2] define regulatory sites for the exonization, alternative splicing, non-coding RNA, transposition, among others mechanisms [3–6]; explained by the *cis*-regulatory motifs included in the Repeat structure [7–9]. Thus, the position, conformation, and motifs define binding sites for proteins or RNA in the formation of regulatory complexes for every active site in the genome like the chromatin remodeling, gene promoters, transposons among other sites [10]. The two primary examples of Repeats with regulatory role are Alu and MIR, both of which are types of SINE-TEs with high density of regulatory binding sites in their sequence (mainly enhancers); It allowed the association of these elements with the control of gene expression in development, stress responses [11], or for the regulation of fundamental processes like the erythropoiesis [12].

The association between Repeats and mechanisms of regulation was proposed by their density in regulatory sites of the genome, and then by ChiP-sequencing of binding sites. Nonetheless, after the study of structural variants in the human genome [13], the feature “specific distribution” of repetitive sequences switched the sense of their density to the structural configuration for precise mechanisms of control, and with a high impact in the genome and phenotype expression[14, 15]. Interestingly, L1 repeats have been described as common and polymorphic LINE-TE elements in the insertion of structural variations among human populations [16], whose variation define human phenotypes, diseases, and the ancestry [17]. Besides, there is evidence of a precise distribution of Repeats for cell functionality and genome organization, for example the Peri-centromeric Repeats in yeast described as higher-order structures to ensure the targeting in chromatin remodeling [18], or the conserved co-localization of specific Alu-MIR TEs among metazoan for the genome architecture [19], or Repeat arrangements defined as makers for embryo development [20]. Thus, Repeats represent no neutral elements, and those have not a stochastic organization or distribution in the human genome [21].

Some mathematical formalisms support this statement as preliminarily we propose in the study of the human genome by nonlinear methods, which allows calculating a significant correlation between Alu density with multifractal dimension [22]. These analyses present quantitative evidence of how the distribution of Repeats affects the genome structure and molecular processes; however, the definition of specific mechanisms requires a more precise exploration beyond their density or variation. We thought that it is plausible to find patterns of Repeats with a cellular sense based on their distribution and configuration in dynamic sites of the human genome. In consequence, here we present the analysis of the upstream promoter regions of the human coding genes to identify these configurations, this approach includes structural descriptions, Repeat and gene clustering, and the integration of functional categories for a co-association among Repeats with cell functions.

## Results

### There are specific configurations of repeats at the upstream promoter region of the human coding genes

**Fig. 1:**
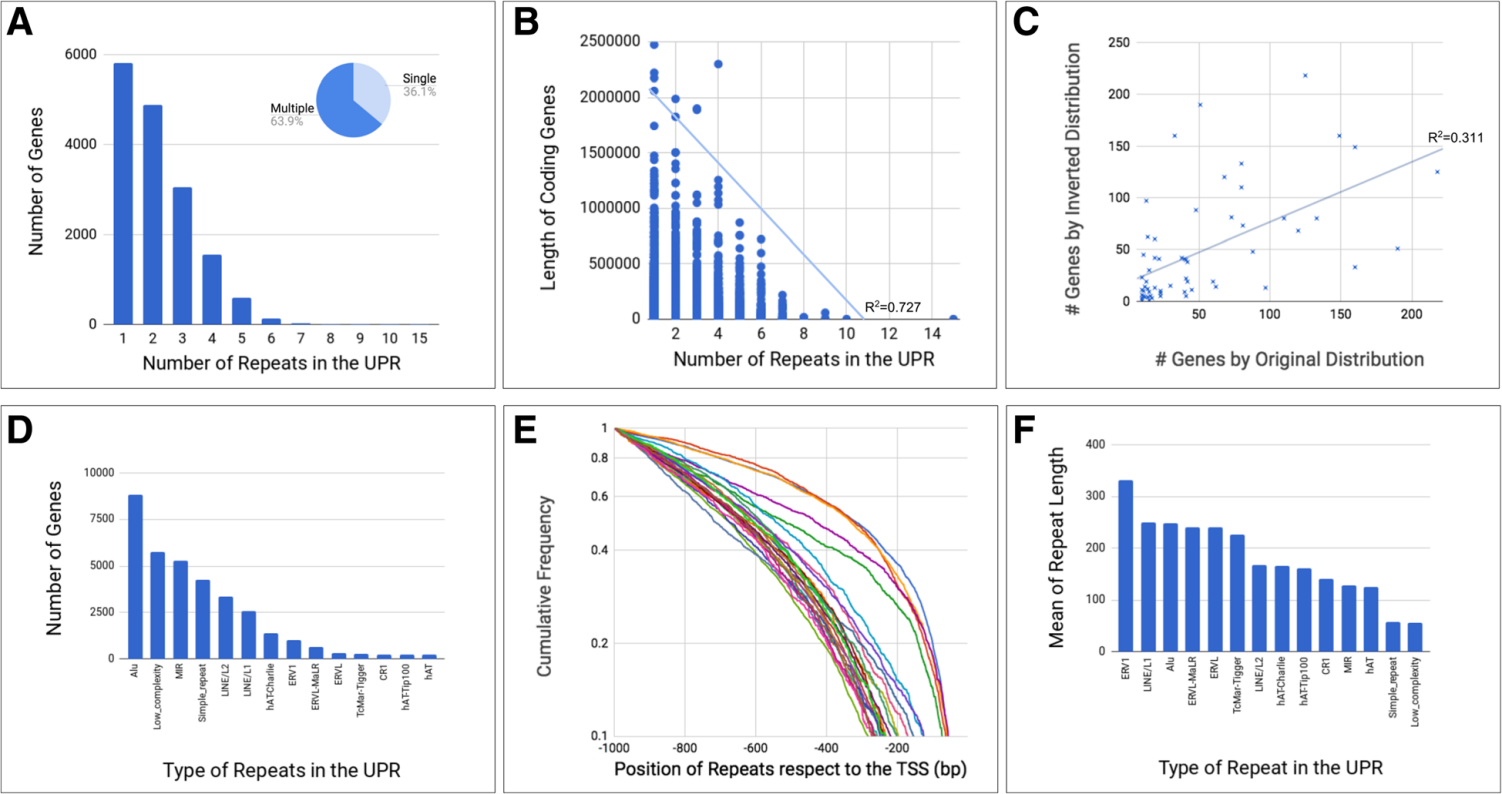
Structural features of Repeats in the upstream promoter region of the human coding genes. **a**. Absolute frequency of genes according to the number of Repeats in the UPR. **b**. Absolute frequency of gene lengths according to the number of Repeats in the UPR **c**. Comparison of patterns with two Repeats at the UPR by using the number of genes at the original and inverted distribution. **d**. Absolute frequency of genes by types of Repeat. **e**. Cumulative frequency of Repeats respect the transcription start sites, and in the range of exploration of 1000 bp, orange, blue, purple and green distant lines represent Simple_Repeats and Low_complesity repeats. **f**. Mean of Repeat length by types

The high density and dispersion of Repeats in the human genome defines regions with regulatory properties for precise mechanisms, which depends on structural regularities in the distribution of those elements in dynamic sites. Therefore, we examined if these Repeats have specific configurations in the range of exploration of 1 kb in the upstream promoter region (UPR) of coding genes; we studied these regions due to the extensive information about models of gene regulation and topological organizations of binding sites. As a result, we found 2729 patterns of specific Repeat configuration in the UPR for 16,091 genes or the ~ 80% of the human coding genes (Additional file 1). We identify that these patterns could include Repeats as single (only one) or multiple (more than one) elements by region, those with a gene proportion of 36.1% and 63.9% respectively, Fig. 1a. Interestingly, the number of Repeats by region inverse correlated with the length of the genes (R^2^ = 0.727), where those with a short length have multiple Repeats and the larger ones a single Repeat in the UPR (Fig. 1b). According to this, we evaluated how the distribution of Repeats could modify their frequency in the UPR. This analysis was necessary to define the significance in the distribution of each Repeat in specific configurations in the upstream promoter region. As shown the Fig. 1c, the correlation between the original and inverted distribution of Repeats in the patterns with two elements is not significative (R^2^ = 0.311), that means the distribution of Repeats is required with a specific configuration in the UPR; and where the order of the components affect their significance in the region. In fact, we found that the original distribution of Repeats has more genes than the inverted one; therefore, there should be some structural feature which defines this distribution.

Commonly, SINE and LINE transposable elements are the Repeats most studied in the gene structure by their high density and binding sites; however, these are only two types of repetitive elements in the genome [1], which means that additional configurations of components could exist in these regions with other molecular properties. In consequence, we analyzed all types of repeats found in the UPR, considering the structural features with the potential to affect their configuration. Thus, 47 types of Repetitive elements were found in the 2729 patterns, which represents more classes than SINE and LINE repeats. In fact, there are Repeats without information about their role in the genome structure (TcMar-Tigger, hAT-Charlie, or CR1), let alone descriptions about their possible regulatory function. The Fig. 1d shows the most “frequent” and “infrequent” elements in the UPR, which includes Alu, Low_complexity MIR, Simple_repeat, LINE/L2, and LINE/L1 as frequent the Repeats; and TcMar-Tigger, CR1, hAT-Tip100 as infrequent. Besides, Alu was found in at least the 54% of the 16,091 coding genes.

Based on this diversity of Repeats, we ask if these elements have the same position in the transcription start sites (TSS) because it is informative about to the alternatives of configuration in the UPR. The Fig. 1e shown the cumulative frequency of the Repeat distribution with respect to the TSS, which presents two significant tendencies: i. for the Repeats Low_complexity and Simple_repeats, whose positions were near to the TSS (~ 50 bp), and ii. for the other set of Repeats with positions after ~ 200 bp. This feature was expected because there is a limit for the molecular interaction between the core promoter and the transcription preinitiation complex; therefore, the diversity of Repeats should be higher after these regions. Besides, the Low_complexity and Simple_repeats are not complex elements in composition or length, therefore their distribution near to the TSS could be a part of the standard sites. To improve these descriptions, we analyze the length of Repeats with the aim to identify if these positions could restrict or correlate with their distribution in the UPR; as shown the Fig. 1f, the lengths of Repeats vary between ~ 50 bp to ~ 300 bp, where the ERV1 (Human Endogenous Retroviral Element) is the longest Repeat, and Low_complexity repeats (polypyrimidine and polypurine repeats) the shortest Repeat. Interestingly, we found no correlation between the length and distribution in the UPR, therefore is plausible to define that the most common Repeats in this region (Alu, Low_complexity, MIR, etc.) have several lengths with specific configurations, whose distribution and arrangement could depend on their potential as structural elements in the gene architecture. This description is more evident with Alu because the length of ~ 300 bp in a range of 1000 bp could be related to a possible topological limitation in their distribution with others; however, we found that the configuration of Alu with other Repeats is highly conserved in the UPR; thus, these patterns should represent conservative and specific sites of combination among Repeats.

### The hierarchical clustering of repeats defines the significance of their configurations

**Fig. 2:**
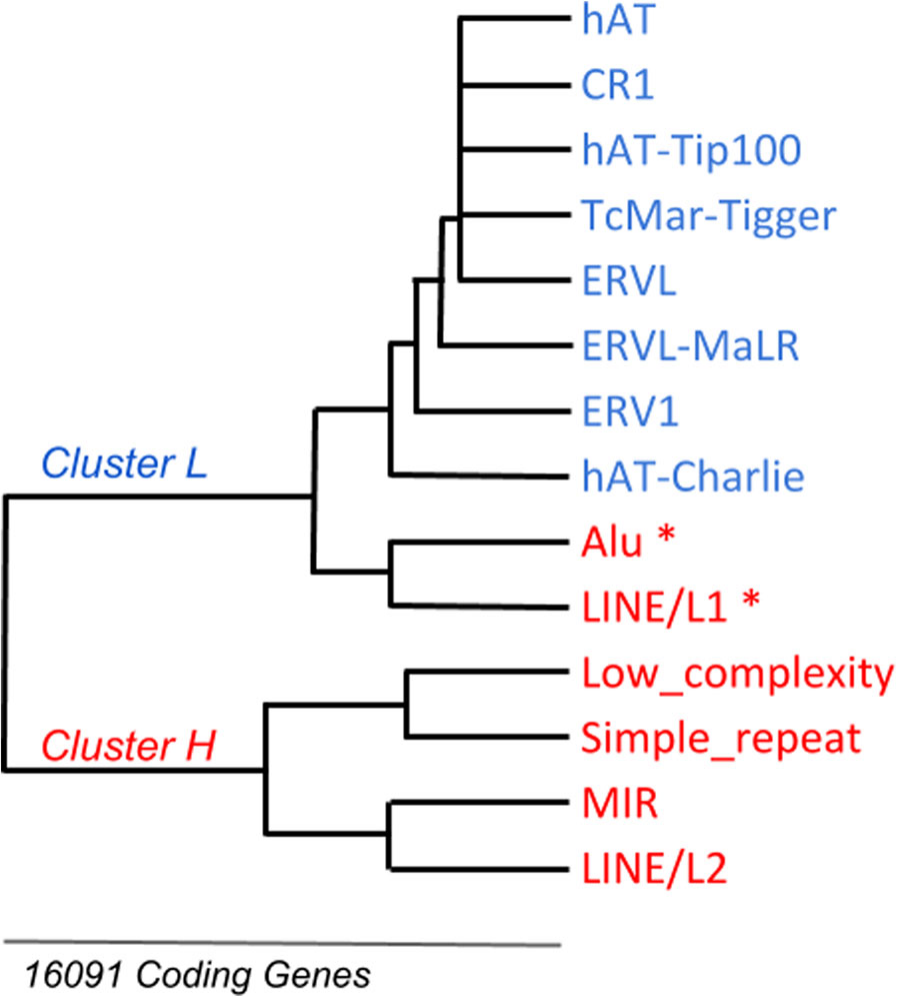
Dendrogram of Repeats based on Hierarchical Clustering Analysis. Colors represent the high (red) or less (Blue) Frequency of Repeats in the UPR. Asterisks represent the exceptions in the tendency of frequent Repeats in subclusters with a high frequency of configuration

The distribution and the specific position of genomic elements in the UPR were very informative for their potential role and configuration; therefore, for a most precise description and significance of these Repeats configurations, we applied hierarchical clustering analysis of Repeats based on their density and co-association in the UPR, which includes the Pearson Correlation Coefficient as measure of similarity (distance) among elements by the clusters. The Fig. 2 shows the dendrogram of the hierarchical clustering, where two clusters were defined based on the frequency of configuration among Repeats, Low (L) and High (H) frequency of configuration in the genes. Interestingly, the cluster H included four of the most frequent Repeats in the UPR (Low_complexity, Simple_repeat, MIR, LINE/L2) and the cluster L the least common (hATs, CR1, TcMar-Tigger, ERVs); nonetheless, there was an exception in the cluster L which included the frequent Repeat Alu and LINE/L1; in fact, these elements were presented in the dendrogram as the basis of several configurations with other infrequent Repeats in the UPR; thus, the distribution of infrequent Repeats have a high frequency of configuration with Alu or LINE/L1 (frequent Repeats).

Additionally, we found that the subclusters for H and L show specific arrangements of Repeats no preliminary reported or associated to configurations but with a significant meaning. For example, the Repeats hAT-Charlie, ERV1, and ERVL-MaLR in the cluster L have been defined individually as ancient Repeats [23]. Similarly, the cluster H presents two subclusters show configurations of short and simple repeats (Low_complexity, Simple_repeat), and other for complex and long Repeats (MIR, LINE/L2); being MIR and LINE/L2 large conserved transposable segments among species [24]. Interestingly, the dendrogram shows a particular distribution among SINE and LINE transposons, where SINE/Alu configure with LINE/L1 and SINE/MIR with LINE/L2. These regularities have been described for Alu<>LINE/L1 for mechanisms of retrotransposition [25]; however, the configuration among MIR<>LINE/L2 has not been deeply explored or associated to molecular mechanisms.

Finally, we examine the specific configurations of Repeats in the UPR with the aim to support this clustering. It was possible by the definition of the nucleotide distances among Repeats respect to the transcription start sites, and the account of genes with the same distribution. The Table 1, shows a fraction of these configurations with the number of genes, where the most frequent patterns were composed by the distribution of the same type of Repeat like Alu<>Alu (439 genes), Low_complexity<>Low_complexity (309 genes), or MIR<>MIR (222 genes). Nevertheless, there were configurations of different types of Repeats like Low_complexity<>Alu (190 genes) or LINE/L2<>Alu (133 genes), with smaller but not insignificant proportion of genes; the full list of patterns is in the Additional file 2. Based on these observations is plausible to define that these configurations, their structural features, and the clustering represent evidence of topological regularities in the upstream promoter region; therefore, the next question was: Do these regularities have functional meaning respect to the gene and cell function?

**Table 1:** Configurations of Repeats in the upstream promoter region of the human coding genes. This table includes 18 of 2729 configurations analyzed in the study

Repeat Configurations	# Genes	# Repeats
TSS<>Alu	1240	1
TSS<>Low_complexity	1062	1
TSS<>MIR	1012	1
TSS<>Simple_repeat	722	1
TSS<>LINE/L2	553	1
TSS<>Alu<>Alu	439	2
TSS<>LINE/L1	359	1
TSS<>Low_complexity<>Low_complexity	309	2
TSS<>hAT-Charlie	236	1
TSS<>MIR<>MIR	222	2
TSS<>MIR<>Alu	218	2
TSS<>Low_complexity<>Alu	190	2
TSS<>ERV1	179	1
TSS<>Low_complexity<>MIR	160	2
TSS<>Low_complexity<>Simple_repeat	160	2
TSS<>Simple_repeat<>Low_complexity	149	2
TSS<>LINE/L2<>Alu	133	2
TSS<>Alu<>Alu<>Alu	126	3

### Configurations of repeats in the UPR have a significant correlation with the cell function

Structural regularities in the genome define functional insights for the cell function; in consequences, the identification of genomic structural pattern should determine functional features at the molecular and cellular levels. Herein, we found several configurations of repetitive elements in the UPR, whose distribution arrayed several genes. Therefore, these sets of genes could have functional association due to the role of Repeats in the promoter structure and the alternative binding sites to a differential transcription factors [26]. Thus, we propose that the Repeats configurations could define the co-association among proteins and pathways for the cell function. We applied a functional analysis based on cell pathway association and functional enrichment analysis for the sets of genes defined by the Repeats configurations. This analysis involved the identification of functional categories over-represented within sets of genes by using two functional databases (Kegg and Gene-Ontology). Kegg was used as a source of curated cell pathways and gene-ontology for the association of cellular components and biological processes.

#### Repeats configurations arrayed essential pathways and cell functions

**Fig. 3:**
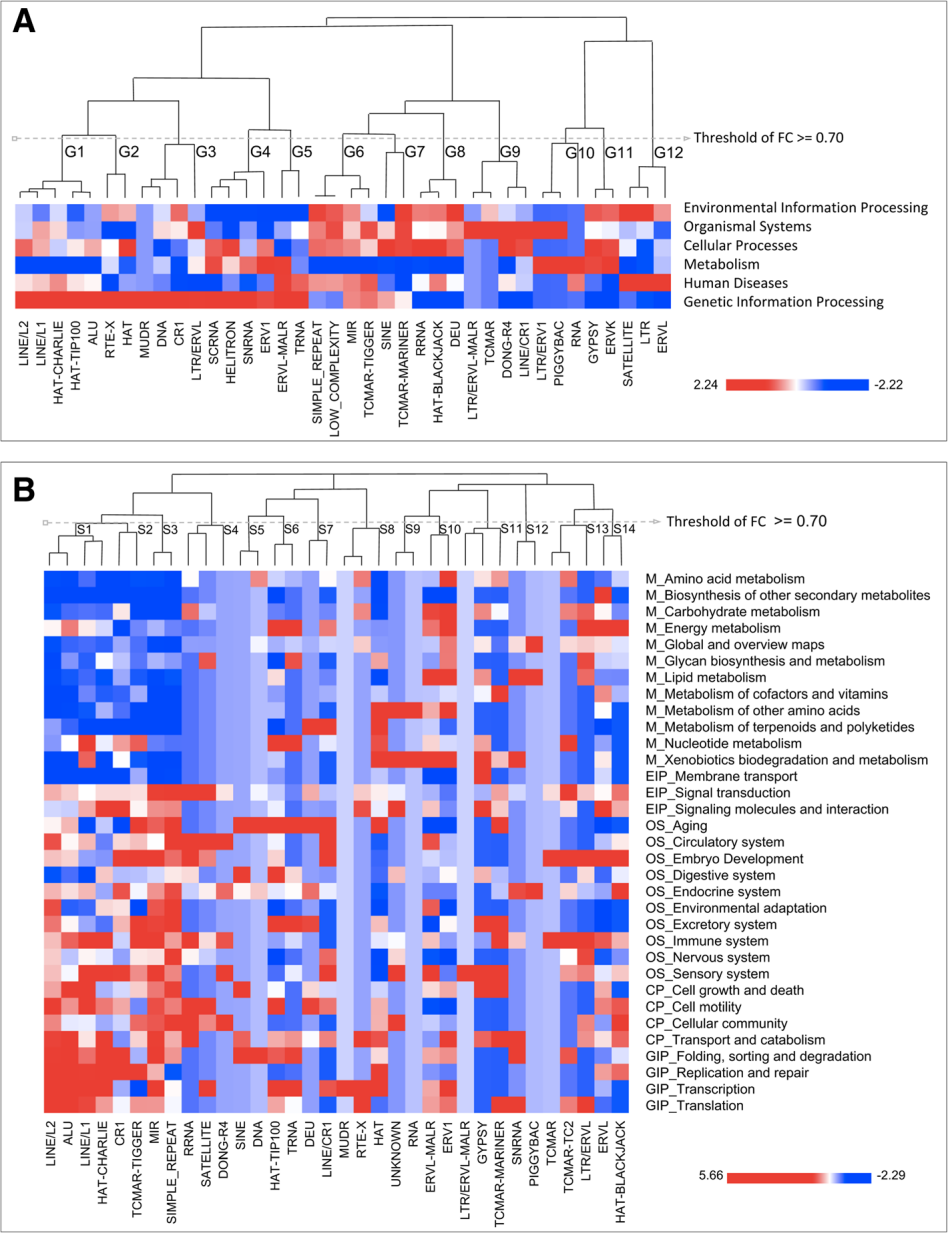
Hierarchical clustering of Repeats based on their frequency in the UPR and a gene value of association to cell signaling pathways. **a**. Hierarchical Clustering for six general categories of pathways, whose minimum frequency of configuration was higher than 0.7 for the definition of subcluster G (General). **b**. Hierarchical Clustering for 33 subcategories of pathways, the same threshold (grey line) was applied for the description of subclusters S (Specific)

The association of cell pathways to the Repeats configurations was possible by the application of hierarchical cluster analysis with complete linkage method, which includes two sets of data in the model: i. The frequencies of each Repeat in the UPR and ii. A functional gene value based on their association with cell signaling pathways retrieved from Kegg. It is important to note, that this value was a constant score based on the normalization of the number of genes by pathway (KO), besides we considered each KO as a curated set of data for a dimensionality analysis. Figure 3 shows two main hierarchical clusters for the cell signaling pathways, which include six general categories (G) and 33 subcategories (S), this analysis includes a heatmap of frequencies with a dendrogram of co-association.

The general categories present 12 subclusters with 0.7 as a minimum Frequency of Configuration (FC) among Repeats, Fig. 3a (See method). Herein, the most outstanding result was found in the categories “*Genetic Information Processing*” and “*Metabolism*”; where clustering of GIP shows five subclusters (G1, G2, G3, G4, G5), that represent a significant number of repeats by category; besides, the distribution of Repeats in each cluster and their frequency defines G1, G3, and G4 as the subclusters with most possibilities of configuration among Repeats by pathway; for example, Alu repeats present a high configuration (FC > = 0.8) with other frequent Repeats like LINE/L1, LINE/L2, HAT-CHARLIE, and HAT-TIP100. About the category of *Metabolism*, the pathways were mainly linked with infrequent Repeats (LTR/ERVL-MALR, TCMAR, DONG-R4, LINE/ERV1, and PIGGYBAC), but with a high grade of configuration in the subclusters G10 and G11; interestingly, in this functional category the most common Repeat was ERV (Endogenous Retroviral Repeat), a family of repetitive elements which includes ERVL-MALR, ERV1, LTR/ERVL and LINE/ERV1; those elements are ancient but fundamental for transcriptional and transpositional activities [27].

Additionally, a relevant result was found in the subclusters G6, G7, and G8 for the functional categories *Environmental Information Processing*, *Organismal Systems,* and *Cellular Processes*; these clusters included three of the most frequent Repeats (SIMPLE_REPEAT, LOW_COMPLEXITY and MIR) configured with infrequent Repeats (TCMAR-TIGGER, SINE, TCMAR-MARINER, rRNA, HAT-BLACKJACK and DEU), which represents different grades of configuration to specific pathways. For example, TCMAR-TIGGER and basic-SINEs have not a significant arrangement for the pathway *Environmental Information Processing*, but those represent a clear pattern for *Cellular Processes*. Based on this finding, we propose that the clustering and the heatmap frequencies represent an overview of configuration among Repeats with cellular meaning in all subclusters; it includes the definition of singularities in essential or interesting pathways like was found in subcluster G12, where the Repeats SATELLITE, LTR, and ERVL configured in many disease-causing genes (*Environmental Information Processing* and *Human Diseases)*.

Based on these findings, we analyzed specific subcategories of pathways for a most precise association between the Repeat configurations to cell functions. The Fig. 3b presents the hierarchical clustering for these subcategories, where 14 subclusters were co-associated to specific pathways; these subclusters include 34 Repeats with an FC higher than 0.7; there were particular configurations with two or three Repeats linked to specific cell processes; for example, the Repeats in the subcluster S1, mainly correlated to *Replication*, *Transcription*, and *Translation*; or the Cluster S10 related to *Metabolic* processes.

Herein, the grade of configuration among Repeats is most diverse than in the general pathway categories; however, the association of subcategories is more precise and assertive respect to the frequency of Repeats. For example, the subclusters S1, S2, and S3 include the most frequent Repeats in the UPR, and their association with functional subcategories is more accurate than other Repeats; in fact, based on these clustering we propose Alu, LINE/L1, LINE/L2 and MIR as the significant Repeats for different grades in the *Genetic Information Processing (GIP)*, *Cellular Processes (CP)*, and *Organismal Systems (OS)* subcategories. Interestingly, these Repeats were less represented in *Metabolisms (M)*; therefore, it was a significant difference between frequent and infrequent Repeats in the UPR. Additionally, we found that the distribution of infrequent Repeats was more specific to some subcategories of pathways, such as the Repeats rRNA, SATELLITE and DONG-R4 (subcluster S4) that were linked to the paths of *OS_Circulatory System* subcategory; or the Repeats HAT-TIP100 and tRNA in the subcluster S6, that were found in six specific subcategories (*M_Energy metabolism, M_Nucleotide metabolism, OS_Aging, OS_Endocrine system, GIP_Folding, sorting and degradation, and GIP_Transcription*); in consequences, the configuration of those infrequent Repeats could be related to new singularities in the UPR of specific genes, and with particular cell pathways (An in-depth experimental validation is required).

Finally, we extracted the most significant information from this hierarchical clustering, with the aim to present the Repeats with the high frequency of configuration by subcluster, and with a functional association to specific categories and subcategories. The Table 2 present three significant clusters where the subclusters S1 and S10 arrayed configurations for specific pathways, which includes the Repeats ALU, LINEL1, LINEL2, and hAT-Charlie for *Genetic Information Processing*; and ERVL-MaLR and ERV1 for *Metabolisms*. Besides, the subcluster S3 arrayed the Repeats MIR and Simple_repeat for multiple but related categories of pathways: The *Environmental Information Processing*, *Organismal Systems*, and *Cellular Processes*. Interestingly, these subclusters of Repeats defined two types of essential cell functions: Pathways to *“Make or Control”* (Subclusters S1 and S10) and pathways to *“Use or Adapt”* (Subcluster S3); therefore, we proposed that: i. The subcluster S1 arrayed Repeats for the most basic and essential molecular processes like *replication*, ii. The subcluster S3 arrayed Repeats for cell responses and environmental adaptation; and iii. The cluster S10 arrayed ancient Repeats for ancient metabolic pathways like *Energy Metabolism.* In consequence, our last question for this work was if these functional insights could be supported by ontological modeling which includes the statistical significance for the functional co-associations.

**Table 2:** Clusters of Repeats with a high frequency of configuration with their specific pathway

Subcluster	Repeats	General Pathway	Specific Pathways
S1	LINE/L2, Alu, LINE/L1, hAT-Charlie	Genetic Information Processing (GIP)	GIP_Replication and repair
			GIP_Transcription
			GIP_Translation
S3	MIR, Simple_repeat	Environmental Information Processing (EIP) Organismal Systems (OS) Cellular Processes (CP)	EIP_Signal transduction
			OS_Aging
			OS_Environmental adaptation
			OS_Excretory system
			CP_Cellular community
S10	ERVL-MaLR, ERV1	Metabolism (M)	M_Carbohydrate metabolism
			M_Energy metabolism
			M_Lipid metabolism
			M_Xenobiotics biodegradation and metabolism

#### Functional enrichment analysis for repeats configurations based on gene-ontology

**Fig. 4:**
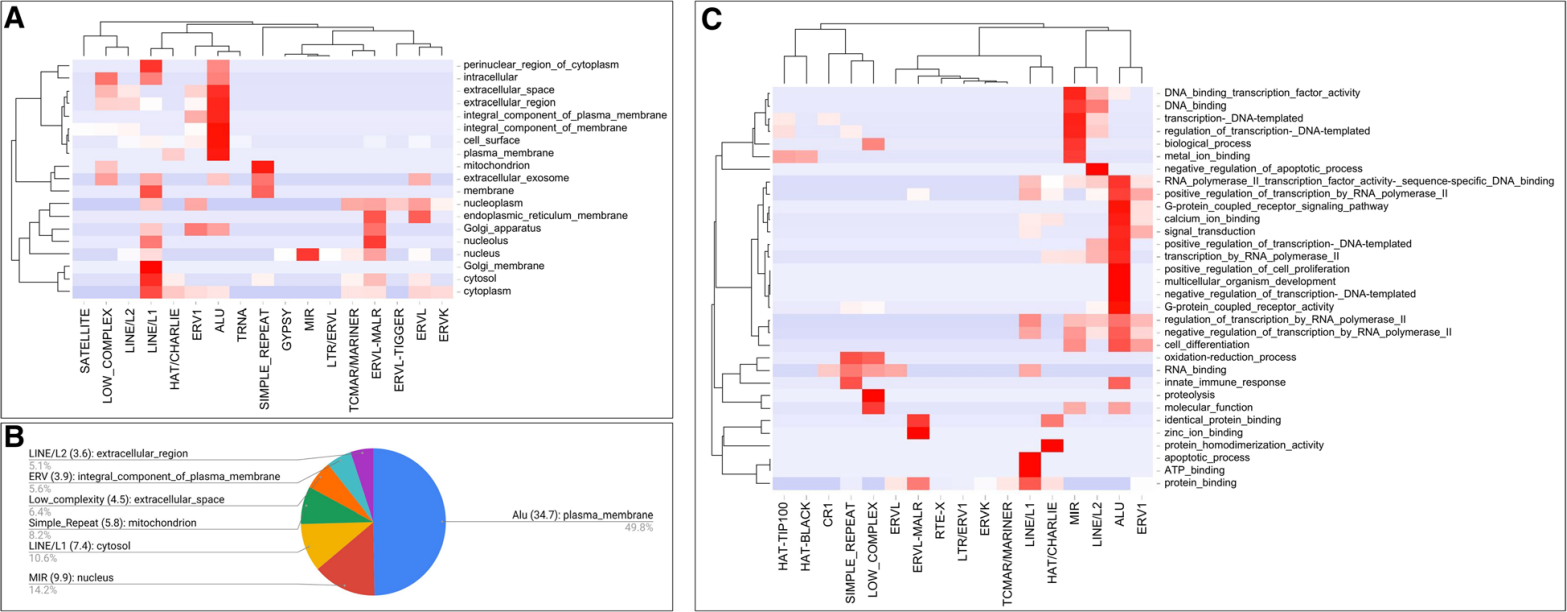
Functional enrichment analysis based on gene ontologies. **a**. Hierarchical clustering of *GO-Cellular-Component*; **b**. Significant compartment by Repeat, where the value in parenthesis represents Log10 (*p*-value). **c**. Clustering of *GO-Biological-Process*. The *p*-values defined the hierarchy and dendrograms the co-associations among categories

Herein we applied a functional enrichment analysis using gene-ontology categories (GO), with the aim to identify regularities in the context of biological modeling. Singular enrichment analysis was applied to the Repeats configurations, where hypergeometric distribution was the statistical method for GO enriched definition, besides we used a restricted cut-off (*p*-value <= 0.05) to restrict the high density of data (16,091 genes, 2729 configurations, and 19,672 genes with GO). Figure 4 presents two hierarchical clusterings for the categories *GO-Cellular-Component* and *GO-Biological-Process*.

Respect to cellular component, the Fig. 4a shows the locations of molecular complexes defined by genes with specific Repeats configurations; these associations allows to specify that patterns based on Alu Repeats arrayed genes with a functional activity in not compartmentalized locations in the cells, which includes cytoplasm, extracellular space, perinuclear region, membrane, or cell surface; in contrast, clusters based on ERVL-MALR, Simple_Repeat or MIR were found in specific organelles like Golgi apparatus, mitochondria or nucleus. Additionally, other Repeats shows different grades of distribution in the whole cell, specifically for LINE/L1 that was distributed in more locations than other Repeat. The significant result of this functional enrichment was the validation of a non-aleatory distribution of Repeats in the UPR and their meaning in the context of cell function, because the p-value for these configurations was less than 0.006; therefore, the Repeats configurations in the UPR of specific genes have a co-association to the distribution of the encoded proteins in the cell locations. In fact, we present in the Fig. 4b how each location in cell correlate with a specific Repeat.

Finally, the *GO-Biological-Process* showed in the Fig. 4c presents the clustering of specific biological functions, where the frequent Repeats MIR, LINE/L2, and ALU were found in particular configurations for specific GO. In this level, Alu was associated with 14 Ontologies of biological processes (p-value less than 0.008), which define several molecular events for cell functionality. Interestingly, each frequent or infrequent Repeat was associated in a higher grade to a particular ontology, for example, infrequent Repeats like ERVL-MALR was related to *protein and zinc ion binding*; and LINE/L2 frequent repeats were associated to *negative regulation of the apoptotic process*. In consequences, these ontologies support the preliminary descriptions that we propose to each Repeat by using Kegg pathways, but here was introduced specific models of functionality where the Repeats configurations define specific and precise processes.

## Discussions

Here, we identify that specific configurations of repetitive elements in the upstream promoter region of the human coding genes have several functional insights. The frequency of configuration among Repeats in hierarchical clusters, the rate of each element in the UPR, and the functional co-associations support their significance in the gene architecture. These configurations will be discussed based on structural features, clustering, and the functional role of their related genes.

*The distribution of 47 Repeat types in the upstream promoter regions of the human coding genes defines several configurations of regulatory elements in the UPR.*

The primary challenge in this study was the definition of the number of patterns because was expected at least ~ 10,000 configurations of Repeats in the range of exploration of 1 kb (47^3^). This number corresponds to three elements with an average of length of 200 bp, and with at least 50 bp of interspace (Based on the structural descriptions Fig. 1e and f). Interestingly, we found 2729 patterns of configuration, that means ~ 5.8 genes by pattern; and where the number of elements was inversely correlated with the length of the genes. This configuration represents grades of Repeat distribution for specific genes, a feature described by the density of the elements [1], but here we include length, position, and clustering as essential structural features to describe their possible role for genes. We propose that Repeats configurations define a non-aleatory distribution in the UPR, which support the definition of Repeats as conserved sites for genome architecture [19], or as dynamic elements for genome variation and adaptation [14]. Currently, some configuration has been described to Alu and LINE/L1 repeats, where specific stem-loop structures allow the mechanisms of transposition [25]. However, here we present a complete catalog of configurations not preliminary reported, and even less with co-association with cell function and pathways. In consequence, our findings show the Repeats as crucial elements for simple and complex arrangements in the UPR; a dynamic site where Repeats have been reported as standard elements [2, 7].

Interestingly, three unexpected findings were found in our study: i. Although the range of exploration was only of 1 kb, there were genes with many Repeats in the configuration, which those includes genes with medical interest like a *Hypertension-Related Calcium-Regulated Gene Protein*, or the *Interferon-Induced Transmembrane Protein 3* (Table 3); ii. The length of Repeats was not directly related to the Repeat frequency or their configuration with multiple elements in the UPR; in fact, the most common Repeats (Alu repeat) are not the shortest element, and their distribution is not structurally restricted; and, iii. There were ancient Repeats as infrequent elements but highly configured in the UPR, such as the CR1 retroposons studied in birds [28], or hAT repeats, a transposon superfamily conserved from plants to animals [29]. These findings suggest that configurations depend on properties in each Repeat, the gene, and even their significance among species.

**Table 3:** Genes with a significant number of Repeats at the upstream promoter region

Name	Gene ID	Function	# Rep	Repeats
S1PR4	GeneID:8698	Sphingosine-1-phosphate receptor 4	15	LINE/L2(1),MIR(14)
FBXL12	GeneID:54850	F-box and leucine rich repeat protein 12	9	Simple_repeat(2),Alu(2),LINE/L1(1),Alu(3),LINE/L1,(1)
PSMF1	GeneID:9491	Proteasome inhibitor subunit 1	9	LINE/L2(1),hAT-Tip100(1),Low_complexity(1),Alu(1),Low_complexity(1),LINE/L1(3),Alu(1)
IFITM3	GeneID:10410	Interferon Induced Transmembrane Protein 3	8	Alu(1),ERV1(7)
ZFP69	GeneID:339559	ZFP69 Zinc Finger Protein	8	Simple_repeat(1),LINE/L2(1),Low_complexity(1),Simple_repeat(2),Alu(2),LINE/L1(1)
SRY-Box 8	GeneID:30812	SRY (Sex Determining Region Y)-Box 8	8	Simple_repeat(1),Low_complexity(3),Simple_repeat(3),Low_complexity(1)
CFAP73	GeneID:387885	Cilia And Flagella Associated Protein 73	8	Alu(1),MIR(1),LINE/L1(1),Low_complexity(2),Alu(1),Simple_repeat(1),Alu(1)
PFN3	GeneID:345456	Profilin 3	8	Alu(1),Low_complexity(1),Alu(1),Low_complexity(1),hAT-Charlie(1),Low_complexity(1),Simple_repeat(1),Alu(1)
COMMD5	GeneID:28991	Hypertension-Related Calcium-Regulated Gene Protein	8	ERV1(1),LINE/L1(2),Alu(1),ERVL-MaLR(1),Alu(1),hAT-Charlie(1),Alu(1)
TOMM5	GeneID:401505	Translocase Of Outer Mitochondrial Membrane 5	8	Alu(1),Simple_repeat(1),MIR(1),Low_complexity(3),Alu(1),LINE/L1(1)

(n) = number of copies

*Genes for essential cell processes have a specific configuration of Repeats, which support the genetic determinism of the human genome.*

The molecular role of these configurations could be explained by the kind of Repeat and their position in the genome, because these elements have several regulatory motifs that directly or indirectly are related to mechanisms of control in genetic or epigenetic levels [3–6], an excellent review of functional descriptions of the regulatory role of Repeats is provided by Chuong et al.; [9]. In fact, Repeats have been clearly described by their structure, composition, length, position, and mechanism of transposition; therefore, all these features should be applied to their configuration in the upstream promoter region. However, the high density of Repeats in the human genome has limited their functional associations at cellular levels; there is no clear correlation between structural configurations among Repeats with functional meaning in the context of the cell functionality or regulation [18]. Some examples like in human cancer shows the variation of transposable elements and their mobility as events of deregulation and genome instability which affect the cell function and induce carcinogenesis [30–32]; and in others studies, the configurations between specific LINE/L1 Repeats was reported in essential cellular processes, like in the nervous system neurogenesis [33]; nonetheless, the diversity of element near to the genes involves several alternatives of association between Repeats and the phenotype; in consequence, the functional analysis of Repeat configurations presented in this study could be the basis for most precise descriptions; it gives insights to support networks of co-association between genes and Repeats. In fact, preliminary studies proposed biological associations to specific Repeats [34, 35]; however, we found that the clustering of pathways and ontologies based on Repeats configuration define most precise co-associations; specifically, herein was presented significant functional statement which describes that the configuration of Alu<>LINE/L1 related to “*Genetic Information Processing*”, MIR<>Simple_Repeat to “*Environmental adaptation and Signal transduction*” and ERV Types to “*Metabolism.*” These findings contribute to explain the expected and predicted multifractal behavior that we proposed for the human genome [22] which defines a genetic determinism in regions with high density of Alu repeats since there is a relationship between the multifractality and the Alu content. Herein, the configuration of Repeats allows to define that these elements require specific distribution in the promoter regions to support the genetics and epigenetics mechanisms, being these finding as a new validation of this nonlinear theory.

## Conclusions

In the current study, we have reported the configuration of repetitive elements in the upstream promoter region of the human coding genes. About 80% of genes have at least one Repeat which defines 2729 patterns. These Repeats configurations were related to specific pathways and ontologies; therefore, we propose the configuration of those elements as constitute patterns in the UPR with several implications for cell function.

## Methods

### Identification and description of the repeat distribution in the upstream promoter region

Bioinformatics protocols were applied for the identification of patterns in the gene promoter regions. The data related to genes and Repeats coordinates were retrieved from ftp://ftp.ncbi.nlm.nih.gov/; Genome-Reference (GRCh38.p7). The identification of configurations of Repeats was made a computational pipeline which selects Repeats overlapped at the upstream promoter regions. The selected Repeats were annotated based on their frequency in the upstream promoter region, besides we retrieved the number of Repeats by chromosome, orientations, Repeat length, gene length, most frequent Repeats, among others. The identification of patterns was defined by mean:

$$ {P}_{cR}=R(sp)-\left( Gene(sp)- RD\right)/ RD $$

Where (*P*_*cR*_) is the pattern value based on the coordinates (c) of Repeats (R); (*sp*) corresponds to the start position, and (*RD*) range exploration (1 kb). In consequence, the definition of Repeats in patterns with specific distributions was defined by the rule: *if P*_*ce*_ *≥ 0* and P_ce_ < 1, and the position of each Repeat was defined by the difference respect to 0. Configurations were characterized based on three conditions: i) number of genes, and ii) number of Repeats by pattern, and iii) Functional categories linked to genes with a pattern.

Cell signaling pathways from the human genome were retrieved from Kegg database (*KEGG FTP Academic Subscription 2015–2016*), besides functional ontologies files were retrieved from Gene-Ontology database. For Kegg databases own python scripts were developed to link pathways, KO signatures, and Gene ID to the Repeat configurations. We used the curated KO for a direct association to the GeneIDs. Then, hierarchical cluster analysis with complete linkage method was applied with two sets of data, the frequency of each Repeat in the UPR and a value of association between genes and functional categories. Normalization of data was applied based on the variance of the number of genes by pathways and the frequency of the Repeat by configuration. Besides, we calculate a Frequency of Configuration (FC) among repeats to restrict the clustering to an FC > = 0.7. This FC value corresponds to a frequency of cluster conservation in the hierarchical clustering, which represents the rate of configuration among Repeats. Additionally, the functional enrichment analysis was applied by using functional ontologies. We calculated the frequency of random functional associations of ontologies by using hypergeometric distribution as statistical method due the sets of genes defined by pattern corresponds to SEA (singular enrichment analysis). In consequence, we calculate a *p*-value by:

$$ P\left(X=k\right)=\left(K\begin{array}{c}\mid \\ {}\mid \end{array}k\right)\left(\left(N-K\right)\begin{array}{c}\mid \\ {}\mid \end{array}\left(n-k\right)\right)/\left(\left(N\begin{array}{c}\mid \\ {}\mid \end{array}n\right)\right) $$

Where (*P*) is the p-value for the probability (X) of a set of genes (k) from a pattern with (K) genes, to have random GO category which contains (n) genes and considering the total number of genes with GO categories (N). If the p-value was <= 0.05 the pattern has an enriched GO annotation. Finally, a matrix of correlation was constructed for hierarchical clustering analysis. It includes the Pearson Correlation Coefficient as similarity measure (distance), and the clusters were represented by dendrograms constructed based on UPGMA method. The Hierarchical Cluster Explorer Tool was used for these procedures [36].

## Additional files

Additional file 1: List of genes with configurations of Repeats in the upstream promoter region of the human genes. (XLSX 321 kb)

Additional file 2: List of Repeat configurations in the upstream promoter region of the human genes, full list. (XLSX 55 kb)

## Abbreviations

CP: Cellular Processes
EIP: Environmental Information Processing
FC: Frequency of Configuration
GIP: Genetic Information Processing
GO: Gene Ontology
M: Metabolism
OS: Organismal Systems
TEs: Transposable Elements
TSS: Transcription Start Sites
UPR: Upstream Promoter Region
